# Supplementary material for: Evaluating the Efficacy and Safety of Hepatitis E Vaccination in Reproductive-Age Women: A Systematic Review and Meta-Analysis
Source: Vaccines (Basel). 2025 Jan 9;13(1):53. doi: 10.3390/vaccines13010053 (PMC11768898; doi:10.3390/vaccines13010053)
Supplement: Supplementary file 1 [file vaccines-13-00053-s001.zip › vaccines-3348807-supplementary.pdf]

Quality assessment

Study 1: Aziz et al.

- **Bias due to Randomization Process**: The study used a cluster-randomized design with blinding, which is appropriate. However, any imbalances in baseline characteristics between groups could increase the risk of bias.
- **Bias due to Deviations from Intended Interventions**: The blinding was maintained effectively, which reduces the risk of bias from deviations.
- **Bias due to Missing Outcome Data**: Comprehensive follow-up procedures were in place, minimizing missing data and thus reducing this bias.
- **Bias in Measurement of the Outcome**: Outcomes were physician-confirmed, ensuring validity and lowering measurement bias.
- **Bias in Selection of the Reported Result**: Results appear to be reported as per the initial study plan, minimizing reporting bias.

Overall Assessment: Low risk of bias

Study 2: Zhong et al.

- **Bias due to Randomization Process**: The study maintained rigorous randomization methods, though being a post-hoc analysis introduces a slight risk if randomization was not balanced for this specific subset.
- **Bias due to Deviations from Intended Interventions**: Blinding procedures were well-maintained, reducing the likelihood of deviations from the intended intervention.
- **Bias due to Missing Outcome Data**: Systematic pregnancy outcome data collection reduces the risk of missing outcome bias.
- **Bias in Measurement of the Outcome**: Measurement bias is minimized due to detailed documentation and physician-confirmed outcomes.
- **Bias in Selection of the Reported Result**: Since it’s a post-hoc analysis, there is a potential for selective reporting, although key outcomes were consistently reported as pre-specified in related documents.

Overall Assessment: Low risk of bias

Study 3: Nesbitt et al.

1. **Selection**
    - Representativeness of the Exposed Cohort: The study was conducted in a high-risk setting for hepatitis E, the Bentiu internally displaced persons (IDP) camp in South Sudan, capturing a representative

sample of exposed individuals (pregnant women receiving the HEV239 vaccine during a mass vaccination campaign).

- Selection of the Non-Exposed Cohort: The non-exposed (unvaccinated) cohort was selected from the same population and matched to the exposed group using age, gestational age, and vaccination propensity score, enhancing comparability.
- Ascertainment of Exposure: Vaccination status was confirmed via self-report and verified with vaccination cards, where available. This approach is thorough, though self-report can introduce minor bias.
- Demonstration that Outcome of Interest was Not Present at Start of Study: All participants were included in the study before their pregnancy outcomes were determined, ensuring that outcomes (fetal loss) were measured prospectively.

Score for Selection: 4/4 stars.

2. **Comparability**

   - Comparability of Cohorts on the Basis of the Design or Analysis: The study used propensity score matching to balance key confounding variables (age, gestational age, and vaccination likelihood), creating a comparable group of vaccinated and unvaccinated pregnant women.

Score for Comparability: 2/2 stars.

3. **Outcome**

   - Assessment of Outcome: Pregnancy outcomes were self-reported but verified against antenatal care records when available, improving the reliability of outcome measurement.
   - Was Follow-Up Long Enough for Outcomes to Occur: Follow-up was conducted to capture pregnancy outcomes up to 28 days post-delivery, sufficient to assess fetal loss rates.
   - Adequacy of Follow-Up of Cohorts: A low attrition rate was maintained with a follow-up procedure for individuals who left the camp. However, complete data for all participants may not be guaranteed due to the nature of the setting.

Score for Outcome: 2/3 stars.

Total NOS Score: 8/9 stars – indicating a high-quality observational study.
